# The effect of metalinguistic awareness on bilingual vocabulary knowledge in Chinese deaf and hard-of-hearing (DHH) students

**DOI:** 10.3389/fpsyg.2026.1827855

**Published:** 2026-05-22

**Authors:** Tongtong Shao, Lulu Chen, Fan Li, Feng Lu, Haomin Zhang

**Affiliations:** 1 Faculty of Humanities and Social Sciences, City University of Macau, Macao SAR, China; 2 Armed Police Hospital of Shanghai, Shanghai, China; 3 Weifang University of Science and Technology, Shouguang, China

**Keywords:** Chinese DHH students, metalinguistic awareness, morphological awareness, phonological awareness, vocabulary knowledge

## Abstract

This study aimed to explore the effect of metalinguistic awareness, especially phonological awareness (PA) and morphological awareness (MA), on the bilingual vocabulary knowledge of Chinese DHH students (*N* = 35, mean age = 15.09 years), after controlling for the effect of non-verbal intelligence. Participants completed measures of non-verbal intelligence, Chinese PA, Chinese MA, and vocabulary knowledge in both Chinese and English. The results showed that PA was mildly related to English vocabulary knowledge (*r* = 0.348, *p* < 0.05), while MA significantly correlated with either Chinese or English vocabulary knowledge (*r* = 0.604, *p* < 0.01; *r* = 0.563, *p* < 0.01). Moreover, Chinese MA showed a strong predicting power for Chinese vocabulary knowledge (β = 0.407, *t* = 2.276, *p* < 0.05), while Chinese vocabulary knowledge exerted a potential predicting impact on English vocabulary knowledge (β = 0.378, *t* = 2.064, *p* < 0.05). Notably, this paper highlighted the important role of MA in Chinese vocabulary learning and the potential impact of Chinese vocabulary on English vocabulary knowledge for Chinese DHH students.

## 1 Introduction

Metalinguistic awareness refers to the ability to reflect on and manipulate the structural features of language (Nagy and Anderson, 1995), mainly including phonological awareness (PA), morphological awareness (MA), and orthographic awareness (OA; Bae, 2015; Gillon, 2004; Koda, 2007; Miguel, 2012). It has been justified to play an important role in improving learners’ vocabulary knowledge within the language (Chen et al., 2008; McBride-Chang et al., 2006; Zhang and Zhuang, 2017), even for deaf and hard-of-hearing (DHH) students (Chan, 2023; Smith et al., 2013; Zhang et al., 2023). Notably, there is even a direct cross- linguistic effect of metalinguistic awareness of one language on vocabulary of the other language (Lin et al., 2018; Wang et al., 2009; Zhang and Koda, 2012). Moreover, an indirect contribution could also be made through metalinguistic awareness or vocabulary competence (Luo et al., 2014; Zhang et al., 2016; Zhang and Koda, 2021). DHH individuals are a heterogeneous group with such diverse cultural values and learning styles that they should be treated with utmost sensitivity (Wang et al., 2018). It has been verified that MA has a stronger predictive power for Chinese word recognition (Chen and Shu, 2001; Kuo and Anderson, 2006), while the English language relies more on PA (Bae and Joshi, 2017; Luo et al., 2014). Participants in this study speak Chinese as their first language and learn English as a foreign language and they tend to have a lower level of language proficiency, so only MA and PA, essential skills for early literacy development, related to Chinese and English, respectively, were assessed for them. More importantly, according to the existing literature, the focus has mainly been on PA and MA in terms of metalinguistic awareness among DHH students. Meanwhile, most studies have mainly targeted English-speaking DHH children (Cho et al., 2024), but limited research has been done in the context of China (Chan, 2023). Therefore, this paper aims to extend beyond the current literature by investigating the effect of metalinguistic awareness, especially PA and MA, on the bilingual vocabulary knowledge of Chinese DHH students.

### 1.1 Metalinguistic awareness in DHH students

Phonological awareness (PA) refers to the awareness of phonological units of spoken language (syllables, onset-rimes, and phonemes; Gillon, 2004; Goswami and Bryant, 1990). It can be assessed by different tasks such as syllable completion, same-syllable judgment, syllable deletion, phoneme judgment, and rime judgment (Gillon, 2004). Morphological awareness (MA) stands for the ability to reflect on and manipulate morphemes, which is about analyzing words into smaller meaning parts such as prefixes, roots, and suffixes (Carlisle, 1995) and is mainly classified into inflectional awareness, derivational awareness, and compound awareness (Chan, 2023), thus assisting learners in understanding morphologically complex words (Kuo and Anderson, 2006). To assess morphological awareness, learners are required to judge semantic or semantic-syntactic relationships that depend on the form of the word or its parts (Berninger et al., 2010).

Compared to their peers with typical hearing, DHH students are likely to have challenges in metalinguistic awareness (Cho et al., 2024; Kyle and Harris, 2010), containing deficient PA (McQuarrie and Parrila, 2009; Miller, 2010; Wang et al., 2018) and delayed MA (Goodwin and Lillo-Martin, 2019; Guo et al., 2013; Zhang et al., 2023). As to PA of non-alphabetic languages like Cantonese, it is worth noting that DHH preschool children with cochlear implants had statistically similar levels of syllable awareness, phoneme awareness, and rhyme awareness with their normal hearing peers. Still, they had poorer tone awareness and phonological knowledge (judgment and repair tasks) than their counterparts. These Cantonese-speaking preschoolers with cochlear implants performed best on syllable awareness, followed by rhyme awareness, and performed poorly on phoneme awareness tasks (Tse and So, 2012), which was also reflected by the research to explore the developmental sequence of PA among DHH students speaking English and wearing cochlear implants (James et al., 2005). In terms of MA in alphabetic languages such as English, students who were DHH exhibited a delay in both printed derivational and inflectional morphology (Trussell and Easterbrooks, 2016). Meanwhile, compared with typical hearing counterparts, they performed worse in English morphological segmentation and meaning recognition associated with various printed morphemes (Gaustad et al., 2002). This finding was also justified by another comparative study (Laçin et al., 2018) in which Turkish deaf individuals had significant limitations in morphological awareness about suffixes compared to peers with normal hearing. Although they suffer from insufficient metalinguistic awareness, DHH students, particularly those using sign language, tend to focus on the form of language, including the correct handshape, movement of orientation (Smith et al., 2013), and rhymes (Miller, 2010; Wang et al., 2018).

### 1.2 The effect of metalinguistic awareness on vocabulary knowledge

According to Nagy (2007), vocabulary acquisition is a metalinguistic process, requiring knowing the pronunciation of the word (phonology), meaning unit (morphology), letter combination (orthography), and so on (Bae, 2015). Vocabulary size is closely related to metalinguistic skills (Altman et al., 2018). Many studies have justified the impact of metalinguistic awareness, particularly PA or/and MA, on vocabulary knowledge (Bae, 2015; McBride-Chang et al., 2006; Nassaji and Geva, 1999; Zhang and Zhuang, 2017). For instance, both PA and MA in English played an independent role in predicting English receptive vocabulary for Korean EFL (English as a foreign language) learners (Bae, 2015). Meanwhile, Nassaji and Geva (1999) justified the contribution of PA and OA to ESL (English as a second language) reading-related abilities, including single-word recognition. As for advanced second-language readers, they may rely more on OA than PA in reading. Based on the research conducted by McBride-Chang et al. (2006) that involved Hong Kong Chinese kindergarten children who were ESL learners, phonological syllable-level awareness was more strongly associated with Chinese vocabulary knowledge, while English vocabulary knowledge highlighted the importance of phoneme onset level. Notably, English PA explained a unique variance in English vocabulary knowledge only, but Chinese MA contributed to Chinese vocabulary knowledge.

Moreover, English derivational awareness, a facet of MA, could strongly predict both ESL breadth and depth (Zhang and Zhuang, 2017). Similarly, Chinese morphological compound awareness played a central role in Chinese vocabulary acquisition (Chen et al., 2008). Regarding Chinese-speaking JFL (Japanese as a foreign language) learners, their Japanese MA had unique contributions to Japanese word learning (Zhang et al., 2024). Additionally, the longitudinal study by Zhang et al. (2016) also revealed that MA could significantly predict lexical inference within languages (English and Malay), and the contribution also became strengthened over time, which was echoed by the finding that English MA was significantly related to lexical inference skills in either Chinese as the heritage language or English as the dominant language (Zhang and Koda, 2021).

### 1.3 The cross-linguistic effect of metalinguistic awareness on vocabulary knowledge

Metalinguistic awareness is a resource that can be transferred from one language to facilitate the development of reading and its related abilities in other languages (Koda, 2008). It was found that metalinguistic awareness, including PA and MA, in one language may directly or indirectly affect vocabulary knowledge of the other language (Bae and Joshi, 2017; Lin et al., 2018; Luo et al., 2014; Ramirez et al., 2010; Zhang and Koda, 2012; Zhang et al., 2016).

The study of Luo et al. (2014) has justified that Chinese PA uniquely predicted English word reading, but no unique prediction was found from English PA to Chinese character reading among Chinese-English bilingual children in Canada. Meanwhile, it did not detect direct cross-linguistic effects of morphological compound awareness on word reading between Chinese and English, both concurrently and longitudinally. Still, the indirect effect of Chinese MA on English word reading was found through English MA. Additionally, in the research of Lin et al. (2018) that involved Chinese children of immigrants in America, Chinese phonological rime awareness had both direct and indirect effects on English word reading. Meanwhile, English phonological phoneme awareness also directly contributed to Chinese word reading. As for advanced Chinese EFL (English as a foreign language) readers in China, Chinese MA could facilitate English vocabulary knowledge directly and indirectly through the mediation of learners’ lexical inferencing ability (Zhang and Koda, 2012). Likewise, dominant-language (English) MA contributed only indirectly to heritage-language (Chinese) lexical inference through heritage-language MA and dominant-language lexical inference (Zhang and Koda, 2021).

In addition, there was also unidirectional cross-linguistic transfer from Korean MA to English vocabulary for Korean ESL (English as a second language) learners in primary school (Bae and Joshi, 2017). Similarly, cross-linguistic transfer of MA was found from Spanish to English word reading, not from English to Spanish word reading among young Spanish-speaking ESL participants in Canada (Ramirez et al., 2010). As for Malay-English bilingual participants in Singapore, their English MA had an indirect effect on Malay lexical inference via Malay MA (Zhang et al., 2016).

### 1.4 The effect of DHH students’ metalinguistic awareness on vocabulary knowledge

DHH individuals have problems with vocabulary, such as limited vocabulary size, deficiencies in certain categories of words, and disabilities in relating inflected and derived forms of words (Gaustad, 2000). Meanwhile, they are inclined to fail to make sense of the written text (Antia et al., 2020; Kyle and Harris, 2010; Miller, 2010) and attain grade-equivalent literacy skills (Laçin et al., 2018; Trussell and Easterbrooks, 2016).

The metalinguistic awareness of DHH students has been justified to be significantly related to vocabulary knowledge (Ching and Nunes, 2015; Cho et al., 2024; Gaustad, 2000) or reading abilities (Dillon et al., 2012; Miller, 2010; Wang et al., 2018). For instance, Cho et al. (2024) have found that all PA, MA, and OA uniquely and independently accounted for the variance of Korean word spelling accuracy for DHH individuals with cochlear implants and hearing aids. The contribution of PA to English vocabulary recognition and reading comprehension was also verified by testing 50 Canadian deaf children using American Sign Language (ASL; McQuarrie and Abbott, 2013). It is echoed by the findings of Wang et al. (2018) who made a comparison study between deaf individuals (using ASL) and hearing individuals to investigate the effect of phonological skills. Besides, the experimental research conducted by Chen (2014) in Taiwan verified that DHH children trained by PA intervention showed greater improvement in either PA or sentence reading performance (related to Chinese word recognition and sentence comprehension) than their peers who received the conventional training curriculum. There were also strong correlations between PA and English reading skills, containing single-word reading, nonword reading, and sentence comprehension among DHH children in America. Meanwhile, such relationships could be mediated by vocabulary knowledge (Dillon et al., 2012). However, the causal relationship between PA and reading comprehension did not exist in the research of Miller (2010), in which OA of prelingually deaf individuals played a central role in the processing of written text.

As for Cantonese DHH children, both PA and MA contributed independently to Chinese word recognition, while semantic radical and MA explained significantly more variance (Ching and Nunes, 2015). It is similar to the English word identification process, in which morphographic analysis (relying on visual channels) could be more efficient than phonological decoding for deaf readers (Gaustad, 2000). The study of Chan (2023) has also provided evidence that both PA and MA could foster vocabulary development of DHH children with hearing devices in Taiwan, and MA significantly contributed to the unique variance in the prediction of Chinese reading comprehension beyond the effect of PA. Notably, the importance of MA for word reading, vocabulary knowledge, and reading comprehension has also been verified by the meta-analysis of Zhang et al. (2023).

It is worth noting that the association between PA and word competence was inconsistent in the current literature (Ching and Nunes, 2015), and research on MA and reading-related abilities containing vocabulary knowledge for DHH students is still limited (Laçin et al., 2018; Zhang et al., 2023). Moreover, most studies mentioned above are based on DHH learners in English-speaking countries or Hong Kong and Taiwan in China, but little research has been focused on the context of mainland China. Chinese, as a logographic language, combines graphs and morphemes, which is different from English as an alphabetic language that maps graphs onto speech sounds (phonemes). In addition, children in mainland China learn Chinese characters based on alphabetic pinyin, while Hong Kong learners follow the traditional direct approach without an alphabetic foundation (Perfetti and Dunlap, 2008). Chinese languages in mainland China, Hong Kong, and Taiwan also involve differences in writing and speaking systems. Therefore, it is worth investigating the vocabulary learning mechanism for DHH students from the context of mainland China.

This paper aims to explore the effect of metalinguistic awareness, including PA and MA, on Chinese DHH students’ vocabulary knowledge by answering the following two questions:

1. Does Chinese metalinguistic awareness affect Chinese vocabulary knowledge in Chinese DHH students, after controlling for the effect of non-verbal intelligence?
2. Do Chinese metalinguistic awareness and Chinese vocabulary knowledge affect English vocabulary knowledge in DHH students, after controlling for the effect of non-verbal intelligence?

## 2 Materials and methods

### 2.1 Participants

Studies involving DHH populations often face challenges related to small sample sizes due to recruitment constraints (Aanondsen et al., 2023, Jønsberg et al., 2025). 35 DHH participants (18 boys and 17 girls) selected from a special-education school with both elementary (15 students) and secondary levels (20 students) in Mainland China took part in the study. Their ages ranged from 9 to 22 years old, with a mean age of 15.09. DHH students tended to start school at a later age than children with normal hearing. They would be assigned to classes based on their enrollment time and academic level, resulting in a relatively wide range of age. Chinese was the language of instruction for all participants, all of whom also attended English courses based on the same series of textbooks. As demonstrated by their English vocabulary results, there was an approximately symmetric distribution, further indicating their comparable levels of English proficiency among individuals. Moreover, 10 participants had severe hearing impairment, while the others had mild to moderate hearing loss. As far as the way of communication, 4 participants primarily used sign language, while they were also equipped with aids for hearing. The other 31 mainly relied on cochlear implants and hearing aids. They were not diagnosed with additional disabilities beyond hearing problems. The informed consent was obtained from the participant’s parents or guardians before the research began, and the ethical application was approved by the Faculty Ethics Committee of the authors’ institution.

### 2.2 Measurements

Five measures were conducted to evaluate participants’ non-verbal intelligence, phonological awareness, morphological awareness, and vocabulary knowledge in Chinese and English. Except for the non-verbal intelligence test adopted from Raven (2011), the other four measures were self-developed for this study based on participants’ textbook materials, classroom teachers’ suggestions, and expert’s guidance.

*Raven Colored Progressive Matrices* is a standardized test to assess one’s non-verbal intelligence through abstract reasoning. It targets children between 5 and 11 years old and individuals with mental and physical impairment (Zhai et al., 2020). The test involves thirty-six items in three groups with colored pictures. Participants are required to complete the missing piece based on the given matrices and choose the correct answer among the given choices. There are 12 items taken from Group A of Raven Colored Progressive Matrices (Raven, 2011) in this task, with 1 point for each, and the maximum score is 12 points.

*Chinese Phonological Awareness Test* contains four parts to evaluate participants’ tone awareness, onset-rime awareness, and phoneme awareness. The first three parts should be completed according to the listening material. In the tone awareness part, participants are supposed to identify the Chinese character whose tone is different from that of the other two. For example, *xüě* *(snow)*, *pǎo (run)*, and *piāo (float)* are displayed as a group, and *piâo (float)* should be selected because of its high-level tone instead of a falling-rising tone. In the onset-rime awareness parts, participants need to judge the similarities and differences between two onsets or two rimes in a Chinese compound word with two characters. For instance, onsets in the compound word ***d**iàn* ***d**çng (electric light)* are the same, but they are different in the word ***c**ún* ***q**ián (saving money)*. Meanwhile, the compound word *d**iàn*** *b**iǎo** (electric meter)* involves different rimes, while the word *x**ìn*** *x**īn*** (*confidence*) contains the same ones. In the phoneme awareness part, ten compound words with two to four Chinese characters are shown for participants to evaluate the accuracy of phonemes. For example, a mistake exists in the three-character word *bìi jí xóng (polar bear)*, for a phoneme is missed in the character *xóng*, which should be x**i**óng. There are 40 items in this task, with 1 point for each, and the maximum score is 40 points. The reliability of this task was α = 0.886.

*Chinese Morphological Awareness Test* contains two parts to examine participants’ morphological recognition awareness and morphological discrimination awareness. In the recognition section, Chinese two-character compound words and their morphological constituents are shown, so participants need to identify their relationship. For example, the pair of words *乐 (joy) and快乐(happy)* are related because the meaning of the morpheme*乐(joy)* can be reflected by the compound word *快乐(happy)*. However, such a relationship does not exist in the pair *开 (open)& 开心(happy)* since they have different references. In the discrimination section, participants should discriminate homographic morphemes among three two-character compound words. For instance, in the group of words *学**者(learner)**, 读**者(reader)**, and 或**者(or)**, 或者(or)* should be identified, for the morpheme 者 in the first two words refers to *a person*, which is distinguished from that in the last one. There are 40 items in this task, with 1 point for each, and the maximum score is 40 points. The reliability of this task was α = 0.931.

*Chinese Vocabulary Knowledge Test* contains three sections to measure participants’ vocabulary breadth and depth. In the breath part, pictures and Chinese compound words should be matched accordingly, such as *木头(wood), 沙发(sofa), and 苹果(apple)*. In the depth section, participants are tested on the spelling and meaning of the words. As to the spelling, they have to identify the correct form of the constituent character, *like花**园**(garden) instead of花**圆**, or **对**联(couplets) instead of **队**联*. Meanwhile, the meaning of words should be judged based on the context of sentences. For example, the word *开关 (switch)* in the sentence *他一进家,就打开了**开关.** (As soon as he entered home, he turned on the switch.)* stands for *the power supply of the appliance* rather than *turning on or off*. There are 40 items in this task, with 1 point for each, and the maximum score is 40 points. The reliability of this task was α = 0.927.

*English Vocabulary Knowledge Test* requires participants to identify the correct picture according to the given words about fruit (e.g., *orange*), animals (e.g*., tiger*), numbers (e.g., *thirteen*), and so on. There are 20 items in this task, with 1 point for each, and the maximum score is 20 points. The reliability of this task was α = 0.890.

## 3 Results

### 3.1 Descriptive statistics and bivariate correlations

Table 1 displays the descriptive results of non-verbal intelligence, Chinese phonological awareness (PA), Chinese morphological awareness (MA), Chinese vocabulary knowledge, and English vocabulary knowledge. The average accuracy rates of measures ranged from Chinese PA (38.85%) to non-verbal intelligence (86.67%). Participants performed well on the assessment of the dependent variables, including Chinese and English vocabulary knowledge, with average accuracy rates of 75.78 and 55.45%, respectively. The standard deviations indicated a relatively large spread in all measurements except for non-verbal intelligence. According to skewness and kurtosis, all variables approximately followed a normal distribution. One-sample *t-*tests further confirmed that all mean scores were significantly different from zero (all *p* < 0.001), with *t* values ranging from 11.2 (CPA) to 49.6 (NVI). The 95% confidence intervals (CI) for the means were relatively wider for CPA (12.64–18.45) and CMA (17.56–24.67), but CVK (27.55–33.08) and EVK (9.09–13.08) exhibited moderate CI. These results collectively suggested that participants demonstrated considerable individual differences in metalinguistic awareness, while they showed average performance on vocabulary tasks. The normality of the distribution supported the subsequent use of parametric statistical analyses.

**TABLE 1:** Descriptive statistics.

Variables (sum score)	N	M	SD	SE	Min	Max	Skewness	Kurtosis	*t*	*p*	CI	
NVI (12)	35	10.40	1.24	0.21	7	12	-0.63	0.22	49.6	< 0.001	9.96	10.84
CPA (40)	35	15.54	8.18	1.38	0	30	-0.46	-0.55	11.2	< 0.001	12.64	18.45
CMA (40)	35	21.11	10.03	1.70	5	36	0.22	-1.19	12.5	< 0.001	17.56	24.67
CVK (40)	35	30.31	7.80	1.32	6	39	-0.19	1.52	22.9	< 0.001	27.55	33.08
EVK (20)	35	11.09	5.61	0.94	1	20	0.13	-1.20	11.7	< 0.001	9.09	13.08

NVI, Non-verbal intelligence; CPA, Chinese phonological awareness; CMA, Chinese morphological awareness; CVK, Chinese vocabulary knowledge; EVK, English vocabulary knowledge; SE, Standard error; CI, Confidence intervals.

A correlational matrix in Table 2 shows the relational patterns. Regarding two control variables, age was related to MA and vocabulary knowledge in both Chinese and English (*r* = 0.533, *p* < 0.01; *r* = 0.557, *p* < 0.01; *r* = 0.555, *p* < 0.01), but non-verbal intelligence was only associated with Chinese vocabulary knowledge (*r* = 0.427, *p* < 0.05). As two facets of metalinguistic awareness, PA and MA had a significant correlation with each other (*r* = 0.495, *p* < 0.01), and PA was mildly related to English vocabulary knowledge (*r* = 0.348, *p* < 0.05). Notably, MA significantly correlated with both Chinese and English vocabulary knowledge (*r* = 0.604, *p* < 0.01; *r* = 0.563, *p* < 0.01). Meanwhile, there was a significant correlation between Chinese and English vocabulary knowledge (*r* = 0.626, *p* < 0.01).

**TABLE 2:** Bivariate correlations.

Variables	1	2	3	4	5	6
Age	-	-	-	-	-	-
NVI	0.332					
CPA	0.156	0.146				
CMA	0.533**	0.287	0.495**			
CVK	0.557**	0.427*	0.254	0.604**		
EVK	0.555**	0.269	0.348*	0.563**	0.626**	

*p < 0.05, **p < 0.01. NVI, Non-verbal intelligence; CPA, Chinese phonological awareness; CMA, Chinese morphological awareness; CVK, Chinese vocabulary knowledge; EVK, English vocabulary knowledge.

### 3.2 ANOVA

Table 3 summarized the univariate ANOVA results examining group differences across five linguistic and cognitive measures among participants. As shown, no significant differences and effects emerged for NVI [*F*(1, 34) = 0.64, *p* = 0.78, η^2^ = 0.23] or CPA [*F*(1, 34) = 0.62, *p* = 0.79, η^2^ = 0.22], both with negligible-to-small effect sizes. By contrast, CMA [*F*(1, 34) = 2.39, *p* = 0.04*, η^2^ = 0.53], CVK [*F*(1, 34) = 3.62, *p* = 0.04*, η^2^ = 0.63] and EVK [*F*(1, 34) = 2.15, *p* = 0.05*, η^2^ = 0.50] yielded statistically significant group differences and large effect sizes. Together, these findings suggested that CMA maybe one of the key factors underlying performance in bilingual vocabulary knowledge.

**TABLE 3:** Univariate ANOVA.

Variable	SS	df	MS	*F*	Sig.	Partial *η^2^*
NVI (12)	12.23	34	1.44	0.64	0.78	0.23
CPA (40)	520.56	34	47.32	0.62	0.79	0.22
CMA (40)	1821.79	34	165.61	2.39	0.04*	0.53
CVK (40)	1310.66	34	119.15	3.62	0.04*	0.63
EVK (20)	542.94	34	49.35	2.15	0.05*	0.50

*p < 0.05. NVI, Non-verbal intelligence; CPA, Chinese phonological awareness; CMA, Chinese morphological awareness; CVK, Chinese vocabulary knowledge; EVK English vocabulary knowledge; SS, Sum of square; df, Degree of freedom; MS, Mean square.

### 3.3 Hierarchical regression analysis and quantile regression analysis

**FIGURE 1:**
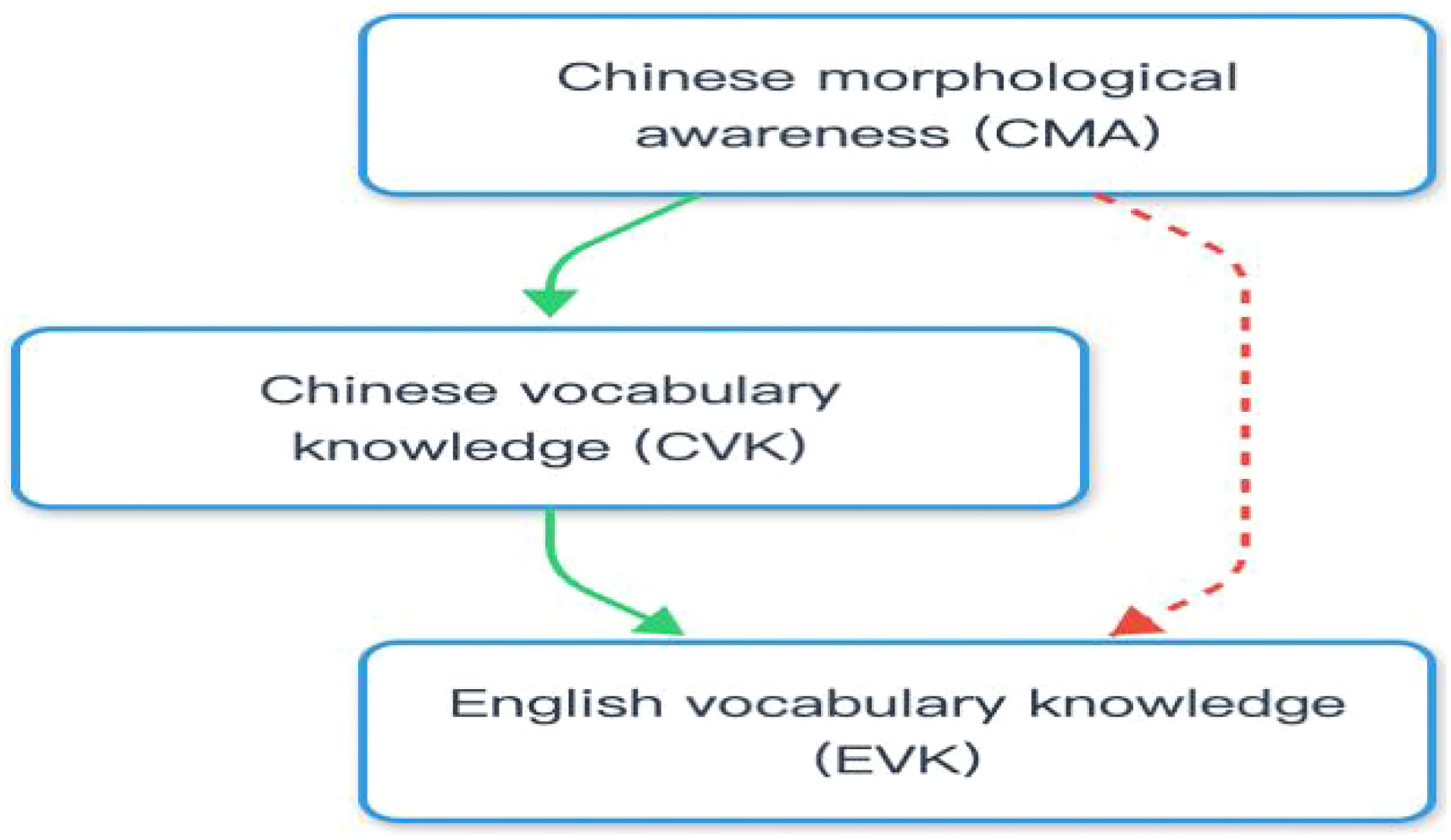
Crosslinguistic path routes (The dashed line indicates an insignificant connection). CMA, Chinese morphological awareness; CVK, Chinese vocabulary knowledge.

Prior to conducting hierarchical regression, key assumptions for parametric statistical methods were carefully examined. Firstly, skewness and kurtosis of all variables demonstrated in the descriptive results confirmed the approximate normality. Secondly, residual diagnostics for both regression models indicated that the residuals were approximately normally distributed, with a mean of 0.000, an acceptable range for standardized residuals (from -3.442 to 1.550 for Model 1 and from -2.129 to 1.692 for Model 2), and a standard derivation close to 1 (0.939 for Model 1 and 0.924 for Model 2). These results collectively justified the use of parametric statistical methods despite the relatively small sample size. Therefore, follow-up regression analyses were conducted to explore the potential contributions of metalinguistic awareness in first-language and foreign-language vocabulary knowledge. Table 4 presented the hierarchical regression results of the effect of Chinese metalinguistic awareness, including PA and MA, on Chinese and English vocabulary knowledge after age and non-verbal intelligence were controlled. In Model 1, MA showed a stronger predicting power for Chinese vocabulary knowledge (β = 0.407, *t* = 2.276, *p* < 0.05). Moreover, Chinese vocabulary knowledge was added as an additional predictor in Model 2, which could explain an 18.3% variance in English vocabulary knowledge. The effect of PA and MA was not found, while Chinese vocabulary knowledge exerted a predicting impact on English vocabulary knowledge (β = 0.378, *t* = 2.064, *p* < 0.05). Therefore, as Figure 1 illustrated, Chinese morphological awareness predicted Chinese vocabulary knowledge, and Chinese vocabulary knowledge played a potential predicting role in English vocabulary knowledge. To put it together, Chinese MA contributed to Chinese VK, which subsequently predicted English vocabulary knowledge. Chinese vocabulary knowledge seemed to serve as a full mediator associating Chinese MA and English VK among DHH students.

**TABLE 4:** Hierarchical regression analysis of Chinese vocabulary knowledge and English vocabulary knowledge.

	Model 1: Chinese vocabulary knowledge		Model 2: English vocabulary knowledge	
	β	*t*	β	*t*
Age	0.269	1.672	0.265	1.573
NVI	0.225	1.601	-0.040	-0.269
CPA	-0.022	-0.145	0.152	0.994
CMA	0.407	2.276*	0.130	0.668
CVK			0.378	2.064*
R^2^	0.486		0.499	
△R^2^	0.110		0.183	
*SE*	5.955		4.300	
△ F	3.195		3.527	

*p < 0.05, **p < 0.01. NVI, Non-verbal intelligence; CPA, Chinese phonological awareness; CMA, Chinese morphological awareness; CVK, Chinese vocabulary knowledge.

**FIGURE 2:**
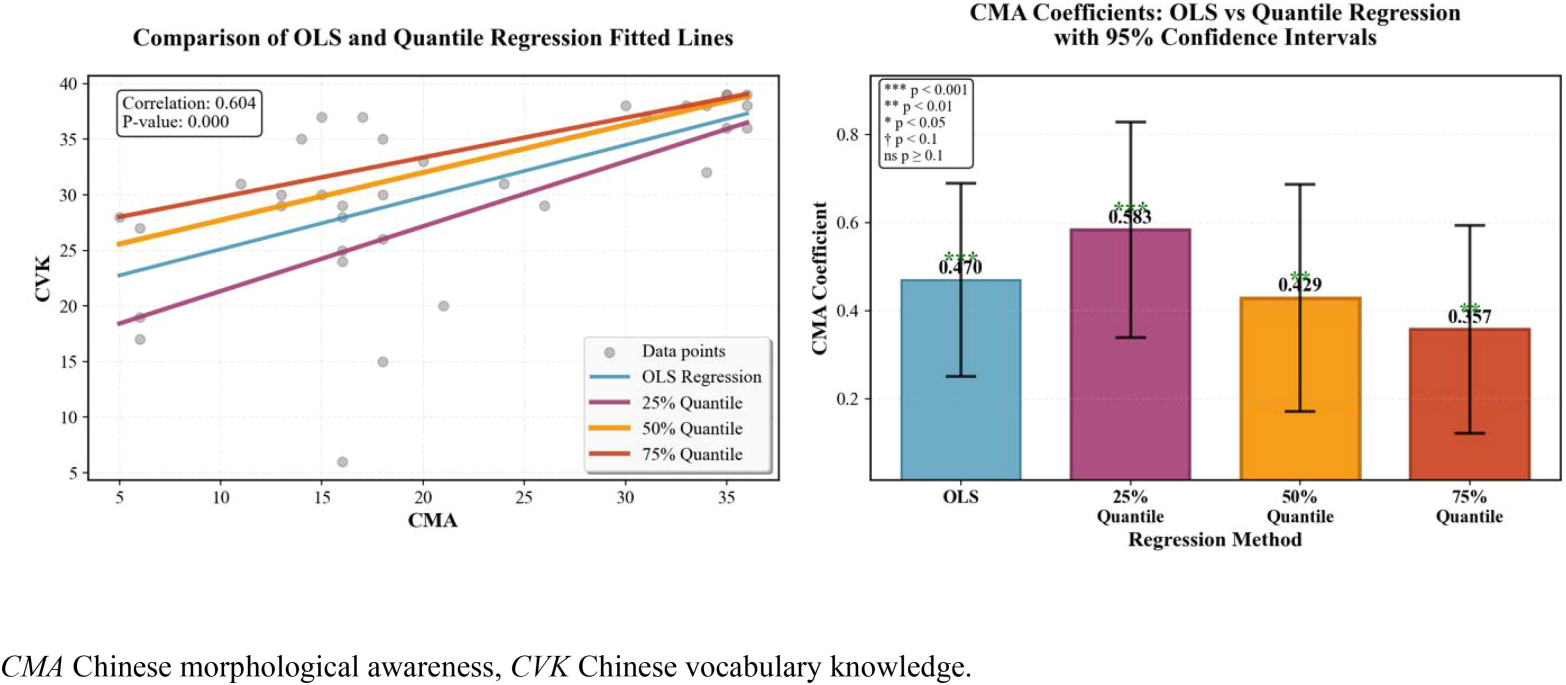
Quantile regression results of CMA and CVK. CVK, Chinese vocabulary knowledge; EVK, Chinese vocabulary knowledge.

**FIGURE 3:**
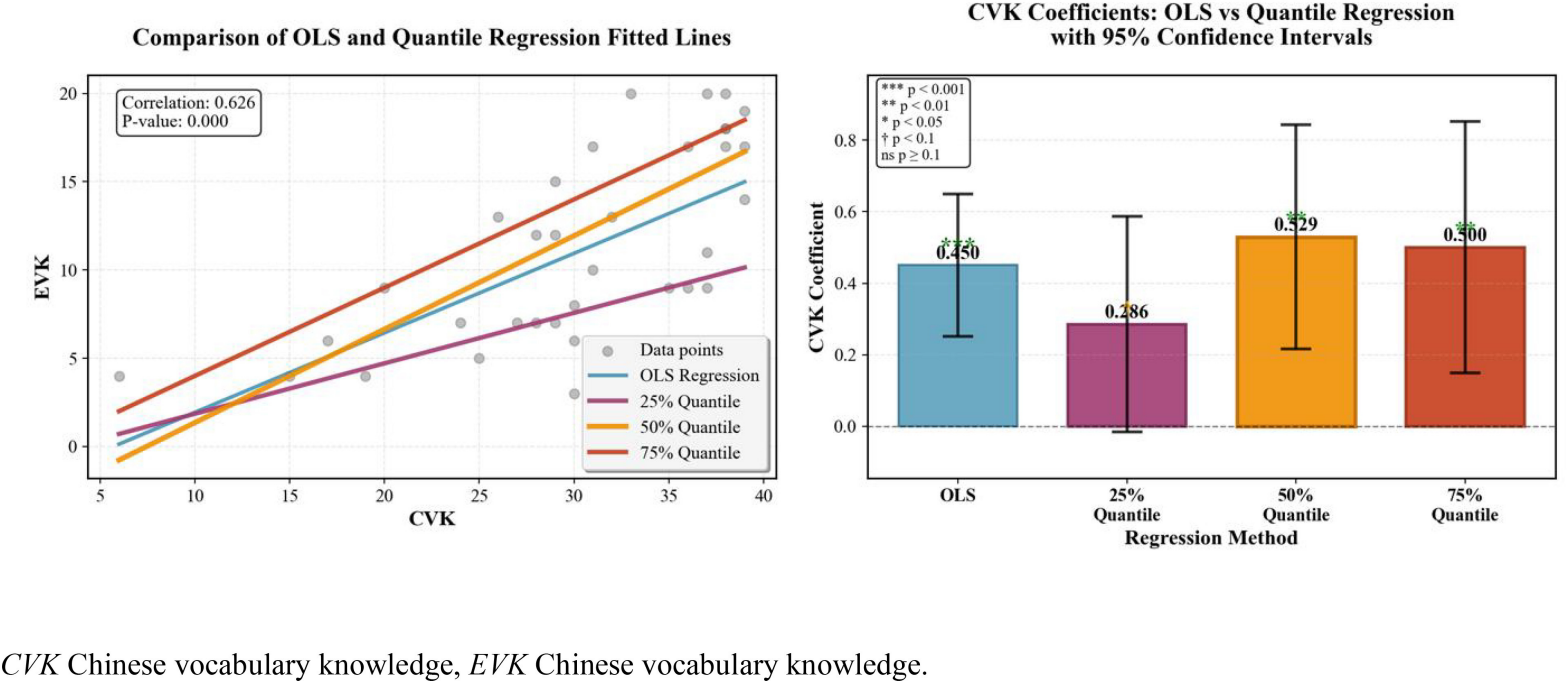
Quantile regression results of CVK and EVK. CVK, Chinese vocabulary knowledge; EVK, Chinese vocabulary knowledge.

Further quantile regression results of Chinese MA on Chinese vocabulary knowledge (Figure 2) showed that Chinese MA could significantly contribute to the Chinese vocabulary of all DHH students, particularly the students who have elementary lexical proficiency. Additionally, as Figure 3 displayed, only DHH learners with intermediate or advanced levels of Chinese vocabulary could benefit their English vocabulary knowledge from their prior word resources.

## 4 Discussion

The current study aimed to explore the effect of metalinguistic awareness, including phonological awareness (PA) and morphological awareness (MA), on vocabulary knowledge for Chinese deaf and hard-of-hearing (DHH) students. Firstly, the results underscored a significantly strong relationship between MA and vocabulary knowledge, which was consistent with the previous findings (Gaustad, 2000; Zhang et al., 2023). Chinese PA was weakly related to English vocabulary knowledge only, but it did not exert any impact on the overall vocabulary competence of DHH students, as hierarchical regression results displayed. On the one hand, children with moderately severe to profound hearing loss did not have comparable speech perception levels with typically hearing children even if they were equipped with hearing aids (Harkins and Bakke, 2011), which hindered the development of PA and reading (Geers, 2003). They tended to make more PA mistakes than MA errors in word spelling that required different skills (Bowers et al., 2014). Based on the descriptive results of all the measures, DHH participants performed worst in the PA test, which justified their deficient phonological decoding ability. On the other hand, some differences exist between spoken Chinese and English, such as syllabic structure, the phonological feature of tone, and homophones (Ching and Nunes, 2015), so it could be hard for DHH students to recognize Chinese characters or words. According to Perfetti et al. (2005), phonological priming effects always occurred earlier than graphic priming effects in the reading of alphabetic languages like English, which was different from Chinese, indicating that Chinese word recognition was less likely to rely on phonological information. It was also supported by the findings that phonological activation did not play an essential role in Chinese character recognition (Chen and Shu, 2001) and Chinese vocabulary (Tong et al., 2018). By contrast, semantic-related factors, such as semantic radical and MA, could compensate for weakness in PA and have a more important influence in identifying Chinese words for DHH individuals (Ching and Nunes, 2015). As far as typical hearing children, MA also had a stronger power than PA in predicting Chinese word identification (Chen and Shu, 2001; McBride-Chang et al., 2003), vocabulary knowledge (Chen et al., 2008; McBride-Chang et al., 2006), and literacy acquisition (Li et al., 2002). For example, in the research of McBride-Chang et al. (2006) involving Hong Kong Chinese children who learned English as a second language, Chinese MA was associated with vocabulary knowledge in Chinese only, showing no correlations with English vocabulary knowledge. Therefore, in contrast with the impact of PA on English word recognition, Chinese PA may not influence vocabulary knowledge to the same extent MA does. Chinese MA can make significant contributions to Chinese vocabulary knowledge for all DHH students, particularly those who have elementary Chinese lexical ability.

Secondly, prior studies have found a significant correlation between MA in one language and vocabulary knowledge in another language, but MA in the first language failed to predict vocabulary proficiency in the second/foreign language (Pasquarella et al., 2011; Tong et al., 2018; Wang et al., 2006). Similarly, as for the participating DHH students, although Chinese (the first language) MA was related to English (the foreign language) vocabulary, it did not show a predicting effect on English vocabulary knowledge. More critically, as Figure 1 displayed, the present study justified the potential mediating impact of Chinese vocabulary knowledge on English vocabulary knowledge for this group of students. Based on the Transfer Facilitation Model proposed by Koda (2008), metalinguistic awareness can be transferred from the source language as a resource to facilitate the development of reading and its related abilities in the target language. However, facilitation through transfer should depend on the sophistication of learners’ metalinguistic insights in the source language and proficiency of both source and target languages (Zhang et al., 2017). DHH participants’ metalinguistic awareness and vocabulary competence in either their native language or foreign language are undeveloped, so the direct effect of Chinese MA on English vocabulary knowledge did not occur. According to the longitudinal research conducted by D. Zhang et al. (2017) that explored cross-linguistic transfer of PA and MA in Malay-English bilingual reading for Singaporean children, neither PA nor MA in one language was transferred directly to facilitate word reading development in the other language. There was also a lack of facilitation effect of English MA on Malay lexical inference in the same context (Zhang et al., 2016). Moreover, it was echoed by the finding that there were no direct cross-linguistic effects of MA on word reading between Chinese and English concurrently and longitudinally (Luo et al., 2014).

More importantly, Chinese vocabulary knowledge played a potential mediating role in predicting English vocabulary knowledge for Chinese DHH students. On the one hand, prior experience can provide resources of knowledge, skills, and abilities for new language learning literacy (Cummins, 1979; Riches and Genesee, 2006), which means that students, including DHH individuals, make use of word proficiency in the first language to facilitate word competence in the second/foreign language. On the other hand, the cross-linguistic influence of MA on vocabulary knowledge in another language can be mediated by the word-level ability of the native language. This can be supported by the research conducted by Zhang and Koda (2021) about college-level participants who grew up speaking Chinese (the heritage language) at home and received English (the dominant language) medium education. The results showed that dominant-language MA was significantly related to lexical inference skills in both languages, but it only indirectly contributed to heritage-language lexical inference through dominant-language lexical inference ability (Zhang and Koda, 2021). By replicating the results from research on typically hearing individuals, this study also justified the same finding that DHH students’ Chinese MA could facilitate English vocabulary knowledge through the potential mediating effect of Chinese vocabulary knowledge. Critically, only DHH learners with intermediate or advanced lexical proficiency can benefit their English vocabulary from their Chinese vocabulary knowledge. Although age was statistically controlled in the analyses, the heterogeneity within the sample could not be fully eliminated. The wide age range of the participants may reflect developmental differences. In other words, age-related development may act as an underlying factor influencing both metalinguistic awareness and vocabulary knowledge. Therefore, the interpretation of these findings should be made with caution.

## 5 Conclusion and future research

The present study explored the effect of metalinguistic awareness containing phonological awareness (PA) and morphological awareness (MA) on vocabulary knowledge of Chinese (the first language) and English (the foreign language) in Chinese DHH students, after controlling for non-verbal intelligence. Compared with the significantly strong relationship between MA and vocabulary knowledge of both languages, PA was only mildly associated with English vocabulary knowledge. Additionally, the hierarchical regression results highlighted the importance of Chinese MA in Chinese vocabulary competence, whereas Chinese vocabulary knowledge played a potential mediating role in associating Chinese MA and English vocabulary competence. These findings underscored the benefits of MA for Chinese vocabulary learning and the potential impact of Chinese vocabulary on English vocabulary knowledge among Chinese DHH students. They can be provided the explicit morphological instruction, such as morphological segmentation or discrimination skills, in Chinese vocabulary learning process. It is more advisable to focus on the morphological processing abilities of learners with elementary Chinese vocabulary proficiency. Regarding English word acquisition, Chinese lexical resources can be fully utilized for DHH students, particularly those who have intermediate or advanced vocabulary levels.

There are a few limitations in this study that need further examination. Given the unique characteristics of the participant cohort, the current research investigated the small sample size of DHH students with a relatively large age span. Moreover, more variables, like English PA and MA, could be involved to explore the cross-linguistic transfer of metalinguistic awareness on the macro metalinguistic level. Finally, even though the variation in English vocabulary performance was moderate and unlikely to substantially confound the observed findings, future research could include a formal measure of general English proficiency as a control variable.

## Data Availability

The original contributions presented in this study are included in the article/supplementary material, further inquiries can be directed to the corresponding author.
